# Editorial: Harnessing autophagy to improve plant quality and resilience

**DOI:** 10.3389/fpls.2023.1266982

**Published:** 2023-08-24

**Authors:** Santiago Signorelli, Kaushal Kumar Bhati

**Affiliations:** ^1^ Food and Plant Biology group, Department of Plant Biology, School of Agriculture, Universidad de la República, Montevideo, Uruguay; ^2^ School of Molecular Sciences, The University of Western Australia, Crawley, WA, Australia; ^3^ Université catholique de Louvain, Louvain la Neuve, Belgium

**Keywords:** autophagy, cellular degradation, stress, plant-pathogen interaction, atg

Plant autophagy, the vacuole-based degradation and recycling of cellular components, is an evolutionarily conserved cellular process. Autophagy is divided into two categories: bulk autophagy (the degradation of nonselective cargoes), which is a major response to the external environment, while selective autophagy (mediated by cargo-specific receptors) is required for normal cellular function and regulation. The machinery involved in plant autophagy has been characterized using cell and molecular biology techniques, and many genetic tools have been developed that have helped elucidate the direct influence of autophagy on plant development, nutrient homeostasis, and stress response. Examples include higher-order mutants for autophagy proteins, visible phenotypes of autophagy-deficient genotypes, fluorescent microscopy, and chemical tools for visualizing autophagy progression at the tissue- and cellular-level. In this Research Topic, we aimed to compile information on works expanding our knowledge on plant autophagy as well as the identification of components involved in the autophagy pathway which could be targeted within biotechnology to address crop production issues (e.g., growth, stress resistance, ripening, and postharvest quality). Six original research papers are presented here. One study confirms the elemental role of autophagy in growth and development in species such as *Physcomitrium patens* (Pettinari et al.). Four works provide new knowledge about the role of genes/proteins in autophagy or senescence such as CV genes in development and responses to environmental stress (Fleitas et al.), a subunit of ESCRT-III complexes in influencing autophagy flux and vacuole morphology (Sun et al.), a Metacaspase from wheat, TaMCA-1d, connecting programmed cell death, stress responses, and autophagy by modulating autophagy-related genes (Yue et al.), and a COPII subunit protein MoSec24B of *Magnaporthe oryzae* in regulating autophagy to govern its pathogenicity on rice (Qian et al.). Finally, one study showed that autophagy is involved in rose peduncle bending (necking), a process that has an impact on the commercial value of the flowers (Lear et al.). Below, we briefly introduce and describe the main findings of these studies.

Necking is a crucial factor limiting vase life leading to a reduction in the quality of the flowers and their commercial value. Although the vase life is known to vary depending on the rose cultivars, pre-harvest and post-harvest conditions, the biological mechanism producing necking is not fully understood. Lear et al., used an RNA-seq-based approach to investigate the biological process involved during rose (*Rosa hybrida* cultivar H3O) necking. At the transcript level, the peduncle bending resulted in the coordinated up-regulation of eleven ATG genes, including isoforms of ATG3, ATG8, ATG11, ATG13, and ATG18. Sugar metabolism and senescence were other processes altered during necking. In particular, the expression of many NAC and WRKY transcription factors was induced, suggesting dehydration-induced senescence by necking. Therefore, the authors suggested that autophagy contributed to the senescence processes. Polysaccharide catabolism was also induced by necking, which was linked by the authors to osmotic stress responses. When necking was severe (> 90°), the expression of trehalose-6 phosphate (T6P) phosphatase G and J was also induced, suggesting a decrease of T6P. Interestingly, T6P was linked as a possible negative modulator of autophagy through SnRK1 inhibition, whereas T6P phosphatases were shown to be positive modulators of autophagy. Therefore, it is not entirely clear whether autophagy was contributing to senescence as proposed by the authors, or mitigating the drought response in response to the changes in sugar levels. In this sense, the authors illustrate an exciting model to explore further the role of autophagy in peduncle necking. Understanding this could lead to strategies in which autophagy is altered to increase vase life and the quality of the flowers.

*P. patens*, commonly known as the moss species *Physcomitrella patens*, is an interesting model system in Plant Sciences due to its evolutionary significance, simple genome, and capacity to efficiently uptake and integrate foreign DNA. These features make *P. patens* an interesting model for gene function analysis and biotechnological applications. Pettinari et al., compared the response of *P. patens* chloronemata and caulonemata cells to autophagy induced-conditions, and investigated the consequences of autophagy deficiency on *P. patens* growth, the development of juvenile protonemata and its transition to the gametophytic phase. The authors showed that *atg5* and *atg7 P. patens* KO-mutants exhibited an exacerbated senescence under low C and N conditions but also presented a senescent yellowish pattern at the centre of the colony (Pettinari et al.), indicating that autophagy has a remarkable role in *P. patens* under normal growth conditions. This role was also evidenced by a reduced size of chloronema and caulonema cells, and caulonema apical cell growth rate under normal conditions. The authors reported elevated levels of indole acetic acid and salicylic acid in the autophagy mutants. After characterizing the expression of *ATG8* isoforms under nitrogen and carbon starvation, and the expression of different ATG genes after leaflet detachment, the authors observed an increased expression of ATG genes in caulonemata than in chloronemata, suggesting a role of autophagy in apical growth, which is consistent with the phenotype observed for *atg* mutants. Moreover, the characterization of autophagic vesicles suggested that autophagy could contribute to the apical growth of protonemata cells during the darkness period occurring under normal growth conditions. Altogether, this work confirms the important role of autophagy under carbon and nitrogen deficiency conditions, but also highlights the remarkable role of autophagy on P. patens growth under normal conditions.

Chloroplast Vesiculation (CV) proteins play a role in a specific chloroplast-degradation vesicular pathway (CVV) during leaf senescence in plants. In particular, the CV proteins participate in the formation of vesicles that encapsulate the components of chloroplasts for degradation, thus playing a role in nutrient remobilization, which can be essential for plant survival under adverse environmental conditions. The specific roles of CV proteins may vary among different plant species and in response to different environmental stimuli. The study presented by Fleitas et al. focused on understanding the role of CV genes in soybean’s response to drought stress. The authors found that the expression of the *CV1* gene was upregulated in response to drought stress, particularly in fast-wilting (sensitive) soybean genotypes, suggesting a negative correlation between *CV1* expression and drought tolerance. However, genes involved in the autophagy-dependent degradation of chloroplast, such as *ATG8* and ATG8-interacting protein 1-plastid-associated bodies (*ATI1*), were induced to higher levels in slow-wilting (tolerant) soybean plants, suggesting a positive role of autophagy under drought conditions. Through the study of the promoters for *CV1* and *CV2* genes, the authors found that *CV1* responded to abiotic stimuli or the presence of stress-related hormones, while *CV2* was mainly active during natural leaf senescence and down-regulated by cytokinin. Interestingly, this study also evidenced the expression of both *CV1* and *CV2* genes in soybean roots, suggesting a putative role of these proteins in root plastids. Overall, the study by Fleitas et al. highlights the differential roles of *CV1* and *CV2* genes in development and responses to environmental stress.

There are emerging pieces of evidence ensuring the extensive crosstalk between the endocytic pathway and autophagy in plant cells. Some studies have shown that components of the endocytic machinery, such as clathrin and Rab GTPases, are involved in autophagy regulation. Additionally, certain endocytic proteins, like the ESCRT (endosomal sorting complex required for transport) machinery, play a role in both endocytic trafficking and autophagy. The investigation by Sun et al. identified a link between ESCRT and autophagy using Moist1 (a subunit of ESCRT-III complexes) from rice blast pathogen fungi *M. oryzae*. The pathogenicity of *M. oryzae* is negatively impacted when Moist1 protein is not functional. The authors reported the developmental impairment along with physiological intolerance to osmotic stresses in these non-functional *ΔMoist1* mutants. Interestingly, the authors reported a higher autophagy flux in *ΔMoist1* compared to the WT using GFP-MoAtg8 marker. The Atg8 marker revealed the altered morphology of the vacuole in *M. oryzae.* The authors argued that this change in vacuole morphology could be due to a defective autophagy pathway. Moreover, it has been proposed that endosomes and multivesicular bodies derived from the endocytic pathway can serve as platforms for the formation of autophagosomes. These compartments may contribute to autophagosome biogenesis by providing membranes or facilitating the recruitment of autophagy-related proteins. Thus, ESCRT complexes and related proteins could indirectly influence the autophagy flux.

Another study in this Research Topic investigates the role of a COPII subunit protein from *M. oryzae* (Qian et al.). To invade plant cells, *M. oryzae* forms a specialized structure called an appressorium on rice leaves. These proteins are secreted by the COPII complex, a protein complex involved in the traditional secretion pathway. It has been shown that the MoSec24-2 protein in the inner COPII coat of *M. oryzae* regulates secretion of effector proteins and auxilin-like proteins. Thus MoSec24-2 is crucial for the pathogenicity of this rice pathogenic fungi. The COPII complex is also linked to autophagy, autophagy is also important for the pathogenicity of *M. oryzae*, and disruption of autophagy-related genes leads to loss of pathogenicity. The specific mechanism by which the COPII complex regulates autophagy in *M. oryzae* is still unclear. This study also identifies MoSec24B, a protein involved in COPII vesicle transport, and explores its biological functions in fungal development and pathogenicity (Qian et al.). The disruption of MoSec24B leads to defects in MAPK signalling, an accelerated fusion of autophagosomes with vacuoles, and reduced appressorium-mediated infection ability. Furthermore, the authors identified the interactions of MoSec24B with other proteins, such as MoRas1 and MoMst50, and their roles in key signalling pathways involved in conidial production, appressorium formation, and cell wall synthesis. The MoSec24B protein is involved in three MAPK pathways and interacts with components of the Mps1 and Osm1 signalling pathways. The disruption of MoSec24B affects cell wall integrity, sensitivity to osmotic stress, and phosphorylation levels of key proteins in these pathways.

MoSec24B plays a role in autophagy and non-functional mutants of this protein showed decreased conidiation, impaired appressorium turgor pressure, and accelerated fusion of autophagosomes with vacuoles. The interactions of MoSec24B with other proteins, such as MoVps27, and their effects on autophagy and pathogenicity are crucial outcomes of this work (Qian et al.). The study suggests that the CWI pathway and autophagy work synergistically to govern the pathogenicity of *M. oryzae*.

Beyond endocytic pathways, Yue et al. reported a new autophagy-stress coping mechanism through cysteine proteases in plants. Metacaspases are crucial proteolytic enzymes that play an important role in programming cell death across plants and some lower eukaryotes. The *Metacaspase 1A* (*TaMCA-1d*) from wheat is expressed at relatively higher levels when young seedlings are exposed to salt stress. The authors used virus-induced gene silencing to knock down the TaMCA-1d mRNA expression. These tissues with silenced *TaMCA-1d* were then assessed for physiological and cellular responses under salt stress. When *TaMCA-1d* was silenced in leaves exposed to NaCl, the authors found higher expression of autophagy-related genes such as *ATG2*, *ATG5*, and *ATG7*. The upregulated autophagy flux was also identified by a higher number of autophagosomes (Yue et al.). On the other hand, these tissues showed low activity for reductive enzymes such as peroxidase and catalase. The lower scavenging of peroxides in the absence of TaMCA-1d results in the activation of program cell death. Overall, metacaspases in plants play a role in connecting programmed cell death, stress responses, and autophagy. They can modulate autophagy by directly cleaving and activating autophagy-related proteins, participating in cargo recognition for selective autophagy, and regulating the activity of key autophagy components. Clearly, it is now important to find additional direct links to fully elucidate how metacaspases interact with the autophagy machinery in plants.

Taken together, these studies evidence different proteins related to autophagy and the importance of autophagy in processes such as plant development, responses to environmental stresses, the control of *M. oryzae* pathogenicity and morphological changes. As climate change and the ongoing deterioration of natural diversity are threatening food and nutritional security across the globe, the identification of potential biotechnological targets of the autophagy pathway and its interaction with stress-specific responses is essential to help the development of novel approaches for climate-smart and nutritional plants.

## Author contributions

SS: Conceptualization, Writing – original draft, Writing – review & editing. KB: Conceptualization, Writing – original draft, Writing – review & editing.

